# A Novel Surface Modification on Core–Shell Yellow Particles for Electrophoretic Display

**DOI:** 10.3390/mi14051063

**Published:** 2023-05-17

**Authors:** Zhi Zhang, Qun Chen, Yao Wang, Guanchen Li, Qingguo Gao, Liming Liu, Jianjun Yang, Xinjian Pan, Feng Chi, Lingling Shui

**Affiliations:** 1 College of Electron and Information, University of Electronic Science and Technology of China, Zhongshan Institute, Zhongshan 528402, Chinaliguanchen0824@163.com (G.L.);; 2 South China Academy of Advanced Optoelectronics, South China Normal University, Guangzhou 510006, China; 3 Gui Yang Institute of Humanities and Technology, Guiyang 550025, China

**Keywords:** electrophoretic particle, organic yellow pigment, core–shell structure, ionic liquid

## Abstract

This paper reports the synthesis of yellow-charged particles with a core–shell structure by modifying yellow pigment 181 particles using an ionic liquid under the sol–gel and grafting methods. The core–shell particles were characterized using various methods, including energy-dispersive X-ray spectroscopy, Fourier-transform infrared spectroscopy, colorimetry, thermogravimetric analysis, and others. The changes in zeta potential and particle size before and after modification were also measured. The results demonstrate that the surface of the PY181 particles was successfully coated with SiO_2_ microspheres, resulting in weak color change but increased brightness. The shell layer also caused an increase in the particle size. Moreover, the modified yellow particles exhibited apparent electrophoretic response, indicating improved electrophoretic properties. The core–shell structure significantly enhanced the performance of organic yellow pigment PY181, making this method a practical modification approach. This method provides a novel way of improving the electrophoretic performance of color pigment particles that are challenging to directly connect with an ionic liquid, leading to the improved electrophoretic mobility of pigment particles. It is suitable for the surface modification of various pigment particles.

## 1. Introduction

Electrophoretic display (EPD) technology is a type of electronic paper (E-paper) display technology with the advantages of low power consumption, high contrast, high viewing angle, and no eye damage [1]. The EPD differs from other display technologies in that its core “electrophoretic ink” quality determines the color and response time of the display [2,3]. Currently, commercial E-paper mainly uses black and white pigments for the electrophoretic display. However, this cannot meet the demand for diversified color display, making color electrophoretic display a focus of E-paper research [4,5,6]. One method to achieve the color display on black and white electrophoretic displays is to use a color filter film. However, this approach has a high light filtration loss, which seriously affects the brightness, contrast, and vividness of the image [7]. Therefore, it is an urgent problem to realize the high-quality color electrophoretic display.

Using color pigment particles to create electrophoretic ink is a feasible solution for realizing the color display in electrophoretic e-paper. These colored particles replace the black and white display particles in electrophoretic display ink, which are controlled by an applied electric field and transferred to form the display [8]. Color pigment particles are typically composed of organic pigments, which offer bright color, rich variety, and low density. Their excellent optical properties make them more suitable for the electrophoretic display than inorganic pigments. However, organic pigment particles are prone to agglomeration under weakening forces between particles and exhibit poor suspension stability, necessitating chemical modification [9,10,11,12]. The key to achieving good dispersion and electrophoretic properties of charged particles in low dielectric constant and surface charge in nonpolar media, and hence the modification of color-charged particles, should focus on improving their dispersion stability and charge capacity [13]. Previous research has achieved a uniform particle size distribution, low brightness, and high reflectivity by grinding yellow pigment PY14 [14]. To improve regularity, yellow 110 was coated with polyethylene and polystyrene microspheres [15]. Additionally, core–shell particles were created by growing nanosized CoAl_2_O_4_ on the surface of SiO_2_ particles, with SiO_2_ as the core and blue cobalt oxide as the shell. The core–shell structure presented various chemical groups that facilitated further modification [16]. However, the core–shell structure can only be completed during the pigment production process and is not applicable to finished color pigments.

The modification of pigment particles has been explored using various methods, such as ionic surfactant, nonionic surfactant, or superdispersant, and reported in previous studies [17,18,19,20,21]. Ionic liquids, which consist of organic cations and organic or halogen anions, are liquid at room temperature and have the ability to easily ionize. It has been reported that ionic liquids can act as charge control agents to modify charged particles [22,23]. Recently, direct adsorption modification of blue pigment copper phthalocyanine using different ionic liquids has been reported, and this method effectively improved the electrophoretic response of the pigment in the nonpolar solvent tetrachloroethylene [24,25]. The simplicity and ease of implementation of this method are noteworthy. However, it should be noted that while ionic liquid is easy to ionize and can improve the Zeta potential of pigment particles after being added to them, it is difficult to directly graft with pigment particles. This limitation restricts the application of ionic liquid in the electrophoretic display. In recent years, the development of color electronic paper has received widespread attention. However, there are few reports on yellow electrophoresis mainly due to the challenges associated with modifying yellow pigment.

This paper presents a novel method for preparing yellow-charged particles, which involves grafting ionic liquids onto colored pigment particles. The resulting yellow-charged particles with PY181 as the core and ionic liquid and SiO_2_ as the shells can be used for the electrophoretic display. SiO_2_ microspheres were utilized to provide abundant hydroxyl groups for the shell layer, while ionic liquid served as a charge control agent to modify the yellow 181 in the core–shell structure and improve the Zeta potential of the particles. The morphological characteristics of the modified yellow particles were also studied, and the changes in the Zeta potential of the charged particles due to the shell structure were analyzed. Finally, the electrophoretic performance of the modified yellow particles was evaluated using an EPD electrophoresis tank. The method is simple to operate and applicable to a variety of finished color pigments, and it is unaffected by the properties of pigment particles, making it a convenient way to enhance the surface potential of charged particles. It provides a new idea for the modification of color-charged particles.

## 2. Experimental

### 2.1. Materials and Methods

Yellow pigment 181 (PY181) was purchased from Clariant Chemical China. Tetraethyl orthosilicate (99%) (TEOS), 1-vinyl-3-butyl imidazole bromide (99%) (IL), OP-10 Emulsifier, tetrachloroethylene (98%), and azodiisobutyroamine (AIBN) were all purchased from Macklin Company, Shanghai, China. Y-methylacryloxy propyl trimethoxysilane (KH570) was purchased from Haifeng Reagent Factory, Binzhou, China. Anhydrous ethanol (99.7%) was purchased from Damao Chemical Reagent Factory, Tianjin, China. Sorbitan monooleate (Span 80) was purchased from Aladdin, Shanghai, China. All reagents can be used without further purification, and ultrapure water is used in the experimental process.

### 2.2. Preparation of SiO_2_-Coated Yellow 181

Silica-coated PY181 particles were prepared using the sol–gel method. Initially, 0.5 g of PY181 particles was dispersed in a solution containing 50 mL of anhydrous ethanol and 5 mL of deionized water. Subsequently, 1 mL of ammonia was added to the solution to adjust the pH to an alkaline level. Ultrasonic dispersion was performed for 10 min. Then, 5 mL of TEOS was added to the solution, which was stirred for 12 h at 30 °C. The mixture was centrifuged and dried, resulting in the production of PY/S particles coated with SiO_2_.

### 2.3. Preparation of PY/S Grafted on IL

In this study, PY/S particles were modified by grafting an ionic liquid onto their surface. Initially, an “intermediate bridge” known as Y-methylacryloxy propyl trimethoxysilane was anchored onto the surface of the PY/S particles. To achieve this, 0.3 g of PY/S was dispersed into 20 mL of anhydrous ethanol and 3 mL of deionized water, and 1.8 g of a silane coupling agent was added. After agitation for 12 h at 90 °C, PY/S–KH570 particles were obtained. Next, 0.2 g of PY/S–KH570, 0.1 g of 1-vinyl-3-butyl imidazole bromide, and 0.03 g of AIBN were added to 30 mL of acetonitrile, and the IL-grafted modified particles were used. The reaction was stirred for 24 h at 100 °C in a high-pressure reactor, and the unreacted materials were removed by centrifugal washing. Finally, PY/S–IL particles were obtained after drying.

### 2.4. Preparation of Yellow and White Dual-Color Electrophoretic Dispersion

About 0.1 g PY/S–IL, 0.1 g titanium dioxide, and 0.1 mg Span 80 were added to 10 mL TCE in the flask. The yellow and white dual-color electrophoretic dispersion was obtained by ultrasonic dispersion for 40 min.

### 2.5. Instruments and Characterization

Scanning electron microscopy (SEM) and energy-dispersive spectroscopy (EDS) (Apreo 2, Thermo Fisher Scientific, Waltham, MA, USA) were used to characterize the morphology, element composition, and size of PY181, PY/S, and PY/S–IL.

PY/S, PY/S–KH570, PY/S–IL, KH570, IL, and potassium bromide powder were dried 6 h in a 70 °C vacuum oven to prepare potassium bromide tablets. The functional groups and chemical bonds of the samples were characterized by Fourier-transform infrared spectroscopy (FT-IR) (irafficity, Shimadzu, Kyoto, Japan) in the wavelength range of 400–4000 cm^−1^ at 24 °C.

An appropriate amount of PY181, PY/S, PY/S–KH570, and PY/S–IL was dispersed into tetrachloroethylene and ultrasonicated for 30 min, and the Zeta potential and particle size were measured by a dynamic light scattering (DLS) spectrometer (Nanobrook 90 plus pals, Brookhaven, NY, USA) at 25 °C.

The colorimeter (Arges-45, Admesy, Ittervoort, The Netherlands) measured the effects of initial PY181, SiO_2_-coated, and ionic-liquid-grafted on the chrominance and brightness of PY181 particles.

## 3. Results and Discussion

### 3.1. Analysis of PY Particles Coated with SiO_2_

**Figure 1:**
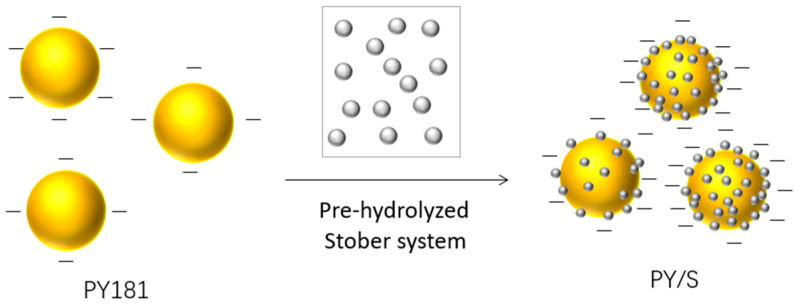
Schematic diagram of the process of silica-coated PY181.

**Figure 2:**
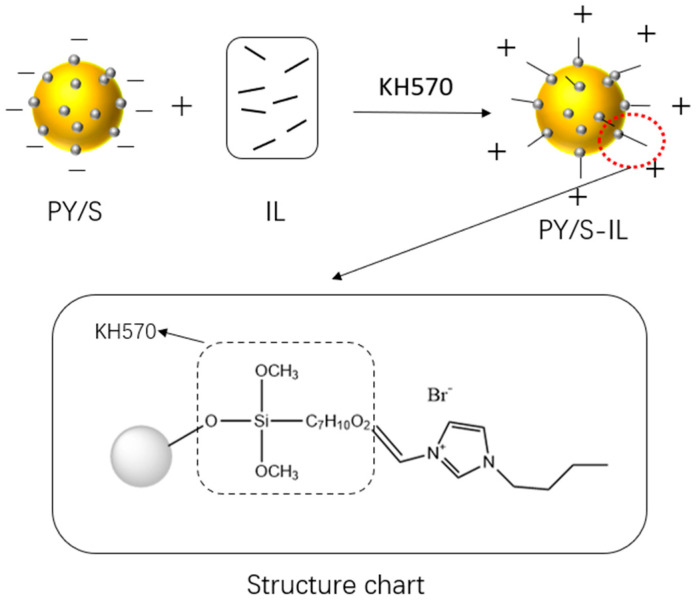
Schematic diagram of the IL grafted onto PY/S.

In this study, the modification process of PY181 is illustrated in Figure 1 and Figure 2. Initially, a Si(OH)_4_ monomer was hydrolyzed by tetraethyl orthosilicate (TEOS), which further condensed to form soluble condensates in the solution. These condensates agglomerated and continued to condense into unstable tiny crystal nuclei. At the same time, these soluble condensates could also condense on the surface of large particles to form microcrystals. In a solution with saturated concentration, tetraethyl silicate hydrolyzed silica, and these nanoparticles were deposited on the surface of the yellow particles to form a silicon shell. The shell exhibited good light transmittance, which had little effect on the chrominance of the yellow particles. Additionally, the electronegativity of the particle surface was enhanced due to the introduction of a hydroxyl group by the silica coating.

It has been demonstrated that the silane coupling agent can dehydrate and condense with the surface hydroxyl groups of silica. As the silica shell on the surface of the yellow pigment provides a large number of surface hydroxyl groups, KH570 can be grafted onto the particle surface as an intermediate. This process plays an important role in the shell formation. Following the successful condensation reaction of the ionic liquid with the silane coupling agent under high temperature and an initiator, the steric resistance of the particles increased, thereby improving their suspension performance. The long side chains of pyrrolidine expanded during the reaction, resulting in the cationic part of the ionic liquid being grafted onto the surface of the particles. This grafting increased the mobility of the particles in an electric field.

**Figure 3:**
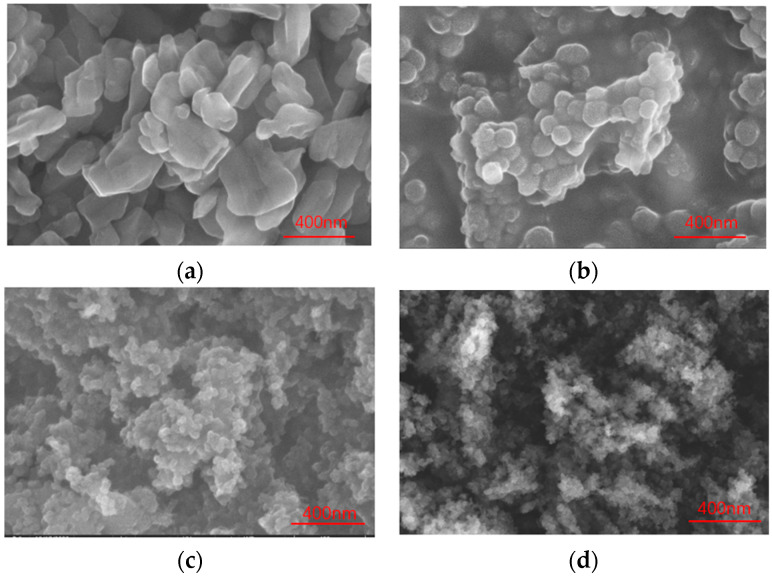
SEM images of PY181 (**a**), PY/S (**b**), PY/S–KH570 (**c**), and PY/S–IL (**d**).

The original PY181 particles, PY/S, PY/S–KH570, and PY/S–IL particles, were dispersed in ethanol using ultrasound for 15 min. The samples were then dropped on the electrode for SEM scanning to observe the changes in particle morphology and coating conditions. The results are presented in Figure 3. Additionally, a dynamic light scatterer was used to determine the mean dispersion size of the particles in ethanol.

The unmodified PY181 particles were observed to be massive particles, overlapping with each other, leading to poor dispersion, with an average particle size of approximately 431.31 nm. After the TEOS reaction, a spherical silica shell was observed, with the shell thickness being affected by the reaction conditions. The nanosilica microspheres did not affect the optical properties of the yellow pigment due to their excellent optical permeability. However, the dispersion of particles after silica coating still required improvement. The average particle size of PY/S particles was approximately 726.56 nm; thus, the thickness of the coating layer was estimated to be approximately 300 nm. After grafting KH570 and IL onto the PY/S particle, especially IL, the distance between the PY/S–IL particles was observed to increase, leading to a reduction in particle accumulation due to charge repulsion. As a result, the aggregation degree decreased, and the dispersion performance increased. The average particle size of PY/S–IL was 682.46 nm.

**Figure 4:**
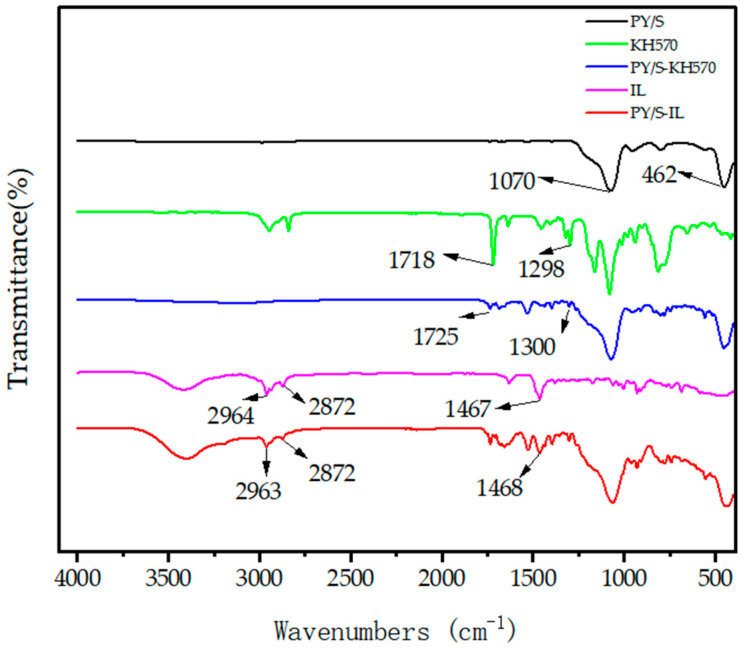
FTIR spectra of PY/S, KH570, PY/S–KH570, IL, and PY/S–IL.

The modification of particles was characterized by FTIR, and the results are shown in Figure 4. The FTIR spectra of PY/S, KH570, PY/S–KH570, IL, and PY/S–IL were analyzed to explore the changes in functional groups before and after particle modification. In the FTIR spectrum of PY/S, characteristic absorption peaks were observed near 1070 cm^−1^ and 462 cm^−1^, which corresponded to the anti-symmetric stretching vibration peak of the Si–O–Si bond and the symmetric stretching vibration peak of the Si–O bond, respectively. The characteristic peaks of KH570 in the FTIR spectrum were numerous and mixed due to its liquid nature. However, characteristic absorption peaks appeared at 1718 cm^−1^ and 1298 cm^−1^, indicating successful reaction with the silica shell’s hydroxyl group and grafting onto PY/S to form PY/S–KH570. The FTIR spectrum of IL showed characteristic absorption peaks at 2964 cm^−1^ and 2872 cm^−1^, corresponding to the tensile vibration peak of C–H in the alkyl; 1467 cm^−1^, representing the plane bending vibration peak of methyl and methylene; and 1632 cm^−1^, which was the C=C stretching vibration peak of olefin. In the FTIR spectrum of PY/S–IL, characteristic absorption peaks of both KH570 and IL were observed, indicating the successful grafting of KH570 and IL onto the PY/S particles coated with SiO_2_.

**Figure 5:**
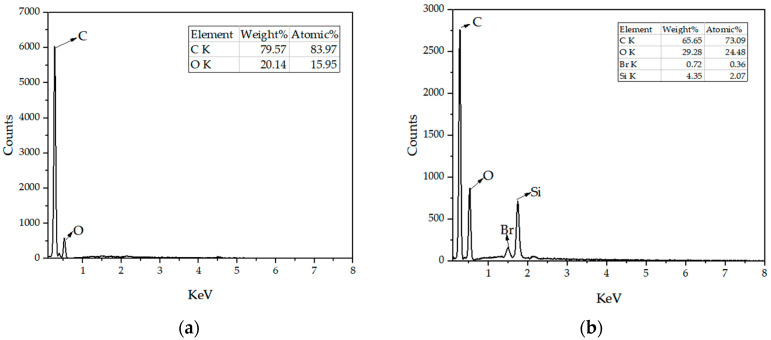
EDX spectrum of (**a**) unmodified PY and (**b**) PY/S–IL.

As shown in Figure 5, the elemental compositions of unmodified PY and PY/S–IL were analyzed by an energy spectrum, and the experimental results of the two samples were compared. Similarly, both samples contained the elements of C and O, and the difference was that, compared with PY (Figure 5a), the apparent energy spectra of Br and Si elements appeared in PY/S–IL (Figure 5b). The appearance of the Si element was due to the presence of SiO_2_ wrapped in PY181, and the presence of the Br element was due to the presence of Br in IL.

The Zeta potential is a crucial indicator for evaluating the performance of charged particles, as it affects their mobility and stability in the dispersing medium. Typically, particles with an absolute Zeta potential value greater than or equal to 30 mV are considered stable. In this study, the modification process’s effect on the particle Zeta potential was investigated using a dynamic light scattering spectrometer, and the results are presented in Table 1. The unmodified PY181 particle had a Zeta potential of −23.98 mV. However, IL had a significant effect on enhancing the Zeta potential of particles. The PY/S–IL particle had a Zeta potential of +42.25 mV, which is 76% higher than that of the original PY181 particle. It is essential to note that the Zeta potential obtained during the measurement is the shear plane electromotive force. The positive and negative values of the Zeta potential in the measuring instrument indicate the direction of particle migration. In this study, the Zeta potential of the modified particles changed from negative to positive, which may be attributed to the long-chain cations of the ionic liquid connected to the particle. The positive Zeta potential of the modified particles indicated that the particles migrated to the negative electrode plate in the electric field, providing a new direction for the yellow particles’ movement in the electrophoretic display.

The core–shell structure’s advantage is that it can alter the movement direction of particles in the electric field. In the context of developing a multicolor electrophoresis display solution, pigment particles with opposite charges can be chosen to form an electrophoresis solution, which can help control the separation of electrophoresis particles in the electric field.

In order to investigate the effect of the core–shell structure on the color of yellow pigment particles, the Admeny colorimeter was utilized to measure the color changes of PY, PY/S, and PY/S–IL. The CIE *x*, *y*, *z* coordinate results are presented in Table 2. It was observed that the brightness increased after coating PY with white SiO_2_, which could be attributed to the highest brightness of white in chromatography, leading to an increase in the brightness of the coated particles. However, the chroma slightly decreased. After the modification with the silane coupling agent and IL, the particle brightness decreased possibly due to the effect of light reflection on the surface of the grafted polymer long-chain coat. The PY/S chroma of the modified particles was similar to that of the coated SiO_2_, indicating that the SiO_2_ microspheres on the surface of PY were still firmly coated on the particles during the grafting of KH570 and IL. The yellow pigment particles of the core–shell structure still maintained a bright color.

### 3.2. Electrophoretic Properties

**Figure 6:**
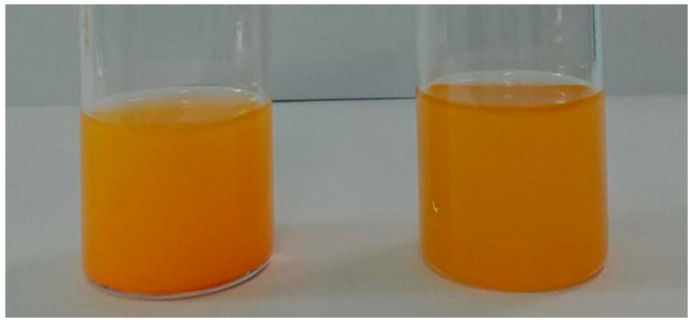
PY181 (**left**) and PY/S–IL (**right**) solutions dispersed in tetrachloroethylene.

**Figure 7:**
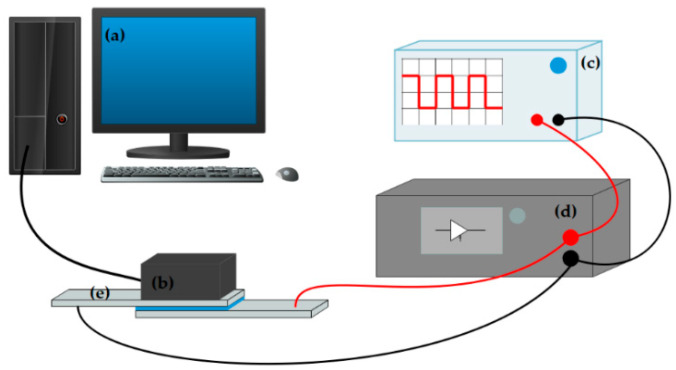
Electrophoresis test platform: (**a**) computer, (**b**) colorimeter, (**c**) oscilloscope, (**d**) voltage amplifier, and (**e**) electrophoresis tank.

In order to evaluate the influence of sol–gel and grafted methods on the electrophoretic performance of PY181 particles, a platform was designed for testing and analyzing the electrophoretic behavior of the particles. This platform consisted of two main parts, namely the voltage-driven part and the brightness change test part, as illustrated in Figure 6. The voltage-driving part included an oscilloscope (AFG3022C, Tektronix, Farmingdale, NJ, USA) and a voltage amplifier (ATA-2022H, Agitek, Brædstrup, Denmark), which could be used to adjust the waveform and output voltage size of the oscilloscope through amplifier amplification. Copper conductive tape was used to transfer the conductive surface of two ITO glasses. The brightness change test part was responsible for monitoring the real-time changes in brightness and chromaticity on the display surface as the particles moved in an electric field. This section consisted of a colorimeter (Arges-45, Admesy, Ittervoort, The Netherlands) and computer software. Figure 7e represents the self-made electrophoresis tank, where the conductive surfaces of two ITO glasses were placed opposite each other with an interval of about 1 mm. AB glue was used to seal around the conductive glass, and a needle opening was reserved for filling the electrophoresis liquid. Two copper tapes were used to connect the ITO glass conductive surface as wires.

Figure 7 illustrates the principle of measuring the electrophoretic properties of particles. Initially, yellow and white particles are irregularly suspended in the solution without applying an electric field, as shown in Figure 7a. When the ITO glass on the upper surface is connected to the positive electrode of the power supply and the ITO glass on the lower surface is connected to the negative electrode of the power supply, the negatively charged white titanium dioxide particles migrate and aggregate upward to the plate, as the upper plate is positively charged. Conversely, the yellow particles, carrying a positive charge, migrate to the downward plate. The white display can be observed directly above the electrophoresis tank, as shown in Figure 7b. When the upper plate is negatively charged and the lower plate is positively charged, the yellow particles move to the upper plate, and the white particles move to the lower plate. The yellow display can be observed on the surface of the electrophoresis tank, as shown in Figure 7c. The colorimeter can measure and record changes in the EPD’s surface brightness and chroma, which are expressed by CIE coordinate changes.

**Figure 8:**
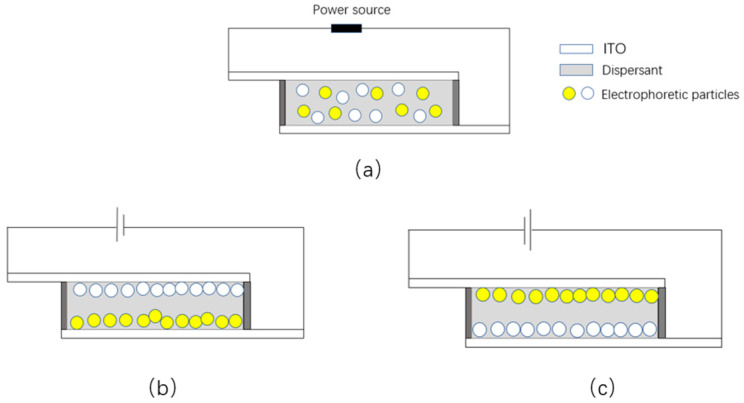
Schematic diagram of yellow–white two-color electrophoretic display: (**a**) no voltage applied, (**b**) a display with positive upper plate, and (**c**) a display with negative upper plate.

In order to conduct the electrophoretic testing, the EPD container was filled with the electrophoretic solution to be tested. During testing, the colorimeter was used to take white as the reference color. A square wave voltage with a period of 5 s and a size of 5 V was selected for the oscilloscope, which was then amplified ten times by the voltage amplifier and applied to the EPD. The data collected by the colorimeter were input into a computer, and real-time changes were observed through Admesy, the software supported by the colorimeter. The results of the test are presented in Figure 8a, where the *x*-axis represents the number of measurement points of the colorimeter, and the *y*-axis represents the surface brightness of the display. When a voltage of +50 V was applied to the upper plate, the white titanium dioxide particle moved upward to the plate due to its negative charge, leading to an increase in the measured brightness of the display, as shown by the rise of the *Y*-axis. At this point, the yellow PY/S–IL particle turned white on the lower plate, as shown in Figure 8b. When the upper plate was negatively charged, the yellow PY/S–IL particle moved up the plate due to its positive charge, leading to a decrease in the display’s brightness, as indicated by the decrease in the *Y*-axis, and the display turned yellow, as shown in Figure 8c.

**Figure 9:**
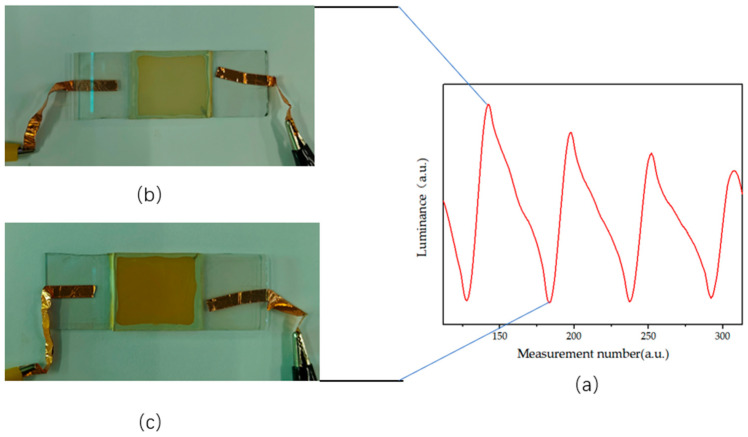
PY/S–IL particle electrophoresis drive test results: (**a**) display brightness change test curve, (**b**) display top view with positive charge, and (**c**) display top view with negative charge.

This measurement results of EPD luminance and chromaticity under the action of applied electric field are shown in the Figure 9. As the test period increased, the amplitude of the brightness variation of the display decreased possibly due to the positive and negative charges between the yellow and white particles canceling out and the electrophoretic power of the particles decreasing. Although the PY/S–IL particle was mixed with white titanium dioxide particles, it could not reach pure white when the upper plate was positively charged during the measurement, appearing as light white. This may be attributed to the smaller particle size of the selected titanium dioxide particle, which was at the micro- or nanolevel, compared with that of the PY/S–IL particle. Additionally, the yellow color saturation was higher than white, so the titanium dioxide particles could not fully cover the yellow particles.

## 4. Conclusions

The modification method used to create the core–shell structure of PY181 was based on the adsorption of silica microspheres on the surface. Additionally, 1-vinyl-3-butyl imidazole bromide was used to effectively disperse the yellow particles in nonpolar media and improve the Zeta potential. This method was suitable for particles that are difficult to directly connect with ionic liquids, thereby enriching the pigments displayed by electrophoresis. The SEM, FTIR, and EDX results confirmed the successful grafting of IL onto the surface of PY, and the modified particles were dispersed with good stability and high positive charge. The presence of ionic liquid increased the Zeta potential of PY particles to +42.25 mV, and the PY181 yellow particles showed an electrophoretic reaction in EPD cells with a bipolar voltage of ±50 V. Furthermore, the unique properties of ionic liquids make them an alternative to some surfactants and an effective charge control agent, as demonstrated by the results.

## Figures and Tables

**Table 1:** Zeta potential of PY181, PY/S, PY/S–KH570, and PY/S–IL.

Sample	PY181	PY/S	PY/S–KH570	PY/S–IL
Zeta potential (mV)	−23.98	−50.22	−7.50	+42.25

**Table 2:** Chroma of PY, PY/S, and PY/S–IL.

Material	Y (Brightness)	x	y
PY	56.35	0.5640	0.4126
PY/S	62.67	0.5248	0.4169
PY/S–IL	61.88	0.5195	0.4242

## Data Availability

Not applicable.
